# Early and recurrent cerebral vasospasms after aneurysmal subarachnoid hemorrhage: The impact of age

**DOI:** 10.1177/23969873231209819

**Published:** 2023-11-01

**Authors:** Bogdana Tokareva, Lukas Meyer, Christian Heitkamp, Rabea Wentz, Tobias D Faizy, Hanno S Meyer, Maxim Bester, Jens Fiehler, Christian Thaler

**Affiliations:** 1 Department of Diagnostic and Interventional Neuroradiology, University Medical Center Hamburg-Eppendorf, Hamburg, Germany; 2 Department of Diagnostic and Interventional Radiology, Katholisches Marienkrankenhaus, Hamburg, Germany; 3 Department of Neurosurgery, University Medical Center Hamburg-Eppendorf, Hamburg, Germany

**Keywords:** Aneurysmal subarachnoid hemorrhage, cerebral vasospasm, endovascular treatment, age dependency, hemorrhagic stroke

## Abstract

**Introduction:**

Cerebral vasospasms remain a strong predictor of poor outcome after aneurysmal SAH. The aim of this study was to describe the time course of relevant vasospasms after aneurysmal SAH and to determine the variables associated with early-onset or prolonged and recurrent vasospasms.

**Patients and methods:**

We conducted a retrospective, single-center study of consecutive adult patients with aneurysmal SAH admitted between 2016 and 2022 at our tertiary stroke center. Relevant vasospasms, defined as vessel narrowing detected in DSA in combination with clinical deterioration or new perfusion deficit, were detected according to our in-house algorithm and eventually treated endovascularly. The primary endpoint was the diagnosis of relevant vasospasms. As secondary endpoints, the time from hemorrhage to the onset of vasospasms and the time from the first to the last endovascular intervention were measured.

**Results:**

Of 368 patients with aneurysmal SAH, 135 (41.0%) developed relevant vasospasms. The median time between ictus and detection of vasospasms was 8 days (IQR: 6–10). Patients with early-onset vasospasms were significantly younger (mean 52.7 ± 11.2 years vs 58.7 ± 11.5 years, *p* = 0.003) and presented more frequently vasospasm-related infarctions at discharge (58.8% vs 38.7%, *p* = 0.03). In 74 patients (54.8%), recurrent relevant vasospasms were observed despite endovascular treatment. Younger age and early onset were significantly associated with longer duration of relevant vasospasms (both *p* < 0.05).

**Discussion and conclusion:**

Younger age was associated with early-onset and longer duration of relevant vasospasms in this study. More frequent clinical and diagnostic follow-up should be considered in this subgroup of patients that are at risk for poor outcomes.

## Graphical abstract

**Graphical abstract:**
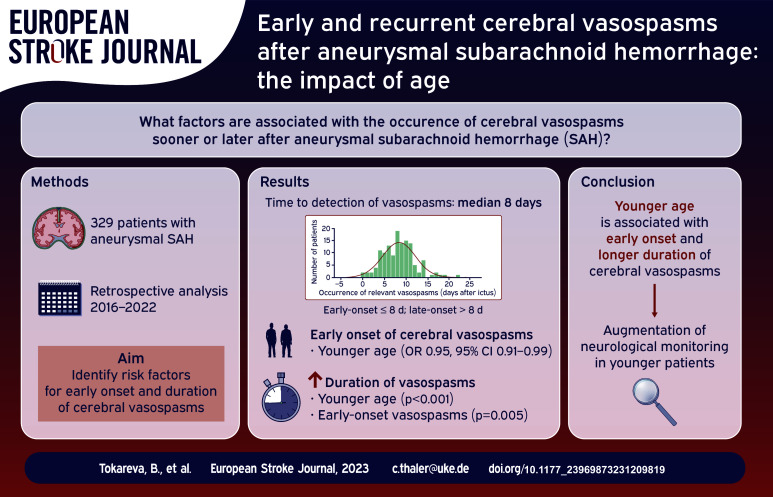

## Introduction

Cerebral vasospasms are a serious but common complication following subarachnoid hemorrhage (SAH). The incidence of cerebral vasospasms after aneurysmal SAH (aSAH) is highly variable, ranging from 9% to 93%.^1^ Despite advances in aneurysm management, cerebral vasospasms remain a strong predictor of poor outcome, attracting research interest over the years.^2^

Although the understanding of the pathophysiology of delayed cerebral ischemia (DCI) has expanded beyond cerebral vasospasms,^3^ a correlation between them remains evident.^4^ Early detection and prompt treatment of vasospasms are crucial in aSAH patients to prevent vasospasm-related infarction and reduce morbidity and mortality.^5^ Especially with emerging treatment techniques, an early detection of cerebral vasospasms seems to be more important than ever.^6,7^

Cerebral vasospasms occur between day 4 and day 14 after ictus, with a peak in frequency on day 7.^8^ More precise data on the risk profile of different patient groups during this time interval are lacking. However, identifying patients who are most at risk on any given day is important for focused screening and timely intervention. This retrospective analysis aimed (1) to describe the time course of relevant vasospasms after aSAH and (2) to determine individual risk factors for early-onset or protracted relevant vasospasms. We hypothesize that younger age is not only associated with occurrence of relevant vasospasms, but also with early-onset and recurrent vasospasms.

## Patients and methods

In this single-center, retrospective study we included all patients who were treated at our tertiary stroke center with aSAH in the time between January 2016 and December 2022. Inclusion criteria were as follows: (1) acute SAH due to a ruptured intracranial aneurysm and (2) age >18 years. Exclusion criteria were (1) SAH due to other causes or without evidence of ruptured aneurysm, (2) death within 14 days after symptom onset without the evidence of relevant vasospasms or vasospasm-related infarction, and (3) missing data. Patients’ demographic and clinical data were retrospectively extracted from the hospital’s digital medical records. The modified Rankin Scale (mRS) was assessed based on neurosurgical or neuroradiological clinical notes at 6-month or last available follow-up.

### Patient management

SAH was diagnosed by computed tomography (CT), magnetic resonance imaging (MRI) or lumbar puncture. After diagnosis of SAH, a digital subtraction angiography (DSA) was performed to confirm intracranial aneurysms. Subsequently, aneurysms were either treated by clipping or endovascularly, depending on the decision of the interdisciplinary neurovascular team.

In cases of extensive SAH or imminent hydrocephalus patients received an external ventricular drainage (EVD). After aneurysm treatment, patients were monitored in the neurosurgical or neurological intensive care unit. To prevent vasospasms patients received nimodipine from the day of admission either orally (60 mg/4 h) or intravenously (1–2 mg/h). The maintenance of an intracranial perfusion pressure of 60–70 mmHg had main priority. Alternatively, a target value of >80 mmHg for the mean arterial pressure was aimed for.

### Detection and management of vasospasms

Daily clinical examinations to detect new neurological deficits and daily transcranial Doppler (TCD) measurements were assessed routinely to screen for relevant vasospasms. TCD ultrasound was performed by experienced physicians or technicians, measuring the mean velocity in the middle cerebral artery and anterior cerebral artery, if possible. According to AWMF guidelines^9^ the TCD measurements were recorded as mean velocity (cm/s). Elevated TCD measurements in the middle cerebral artery were defined as either a mean velocity >140 cm/s or doubling within 24 h. If a new neurological deficit, a decline of consciousness or increased TCD measures were detected, patients underwent additional imaging studies, such as CT angiography (CTA), in most cases with CT perfusion (CTP), or DSA to confirm relevant vasospasms.

Intubated, sedated or not fully oriented patients, who could not be sufficiently monitored clinically, also received daily TCD measurements and baseline CTP within 24 h after aneurysm treatment. In selected cases with normal TCD measures CT imaging studies including CTA and CTP were performed 4–6 days after admission to detect pronounced vessel narrowing or perfusion deficits. If necessary, this was repeated 10–14 days after admission.

If relevant vasospasms were confirmed, patients received endovascular treatment including intraarterial administration of nimodipine (routinely injected in the ipsilateral internal carotid artery of the affected territory or vertebral artery in case of relevant vasospasms of the posterior circulation) or percutaneous transluminal angioplasty via balloon or temporary stent of the affected vessel segment. The flow chart in Supplemental Figure 1 demonstrates the standard operation procedure of our hospital to detect and select patients with relevant vasospasms who are eligible for endovascular treatment. In summary, relevant vasospasms were defined as (1) considerable vessel narrowing in DSA that was consistent with clinical deterioration, defined as the occurrence of focal neurological deficit or a decline of two points or more on the Glasgow Coma Scale,^10^ which could not explained by other causes or (2) considerable vessel narrowing in DSA in combination with a new perfusion deficit in CTP in the affected vessel territory.

### Primary and secondary end points

The primary outcome was the occurrence of relevant vasospasms. The secondary outcomes included the time point from symptom onset, that is, time point of aSAH, to the diagnosis of relevant vasospasms and the duration of relevant vasospasms. Further, the CT images at admission were reviewed by two neuroradiologists with 10 and 4 years of experience, blinded to the patients’ characteristics and outcome, to assess the modified Fisher grade and the incidence of additional intracerebral hemorrhages (ICH). Additionally, CT or MRI after endovascular or neurosurgical treatment and at discharge were reviewed to detect vasospasm-related ischemic lesions. Vasospasm-related infarctions were defined as new ischemic lesions at discharge in hemodynamic or territorial distribution that were not associated with other causes (e.g. treatment-associated, herniation, etc.). Discrepancies were resolved by consensus.

### Statistical analysis

Data were tested for normality and homogeneity of variance using histogram plots and the Shapiro-Wilk test. Descriptive statistics are presented as frequencies and proportions (percentages (%)) for categorical variables and compared with χ2 test, mean (standard deviation (SD)) for continuous normally distributed variables and compared with the unpaired *t*-test, and medians (interquartile range (IQR)) for non-normal continuous variables and compared with the Mann-Whitney *U*-test, respectively.

Binomial logistic regression analysis (logistic regression, stepwise backward, Wald model) was used to assess risk factors associated with early vasospasms and repeated endovascular treatment. Second, we performed multivariate regression assessing variables to predict the duration of relevant vasospasms. Any variable considered clinically meaningful were adjusted for the regression analysis. A *p*-value <0.05 was considered statistically significant.

### Data availability

The data that support the findings of this study are available from the corresponding author upon reasonable request.

## Results

### Study population

Overall, 368 patients were diagnosed with SAH due to a ruptured aneurysm between January 2016 and December 2022. Twenty-one patients died within 14 days after symptom onset without the evidence of relevant vasospasms or vasospasm-related infarction and had to be excluded. Another 18 patients without evidence of relevant vasospasms were excluded due to missing imaging data at discharge.

In 135 (41.0%) patients relevant vasospasms were detected according to our in-house diagnostic algorithm and endovascular treatment was performed. Additionally, 15 (4.6%) patients showed vasospasm-related infarctions in their images at discharge but were not detected by our in-house diagnostic algorithm. Overall, 74 patients (22.5%) presented with a new infarction at discharge that was not present after the treatment. In the patient group with relevant vasospasms or vasospasm-related infarctions, 54 patients (36.0%) received an MRI at discharge, compared to 94 patients (52.5%) in the group without detected relevant vasospasms or vasospasm-related infarctions. Patients with detected relevant vasospasms or infarctions associated with vasospasms had higher rates of ICH at admission and received more likely an EVD. Further, they had a higher Hunt and Hess grade and modified Fisher grade at admission.

In 288 patients the functional outcome could be assessed based on neurosurgical or neuroradiological clinical notes at 6-month or last available follow-up. Patients with relevant vasospasms or vasospasm-associated infarctions presented with a worse functional outcome at follow-up (median mRS 0 vs 3, *p* < 0.001). A detailed overview of the study population compared by relevant vasospasms is displayed in Table 1.

**Table 1.** Demographic and clinical parameters of patients with aSAH.

	All patients *n* = 329	No relevant vasospasms *n* = 179	Relevant vasospasms *n* = 150	*p*-Value
Baseline characteristics				
■ Age (years), mean (±SD)	56.6 (13.1)	57.2 (14.1)	55.9 (11.8)	0.34
■ Female sex, n (%)	222 (67.5)	117 (65.4)	105 (70.0)	0.41
■ BMI (kg/m²), mean (±SD)	26.36 (5.2)	26.4 (5.6)	26.3 (4.8)	0.80
■ Diabetes, n (%)	5 (1.5)	3 (1.7)	2 (1.3)	1.00
■ Hypertension, n (%)	143 (43.5)	74 (41.3)	69 (46.0)	0.37
■ Smoker, n (%)	110 (33.4)	55 (30.7)	55 (36.7)	0.24
Aneurysm location, n (%)				
- *Anterior circulation*				
■ ICA	80 (24.2)	43 (24.0)	37 (24.7)	0.89
■ MCA	46 (14.0)	22 (12.3)	24 (16.0)	0.33
■ ACA	152 (46.2)	83 (46.4)	69 (46.0)	0.95
*- Posterior circulation*	59 (17.9)	31 (17.3)	28 (18.7)	0.75
Imaging details				
■ Modified Fisher grade, median (IQR)	3 (2–4)	3 (1–4)	4 (3–4)	**0.001**
■ ICH, n (%)	102 (31)	41 (22.9)	61 (40.7)	<**0.001**
■ Vasospasm-associated infarction, n (%)	119 (36.2)	-	74 (49.3)	
Clinical deficit				
■ Hunt and Hess grade, median (IQR)	2 (2–4)	2 (1–3)	3 (2–4)	**0.009**
Treatment details, n (%)				
■ Clip	78 (23.7)	40 (22.4)	38 (25.3)	0.53
■ Endovascular	252 (76.6)	139 (77.7)	113 (75.3)	0.62
■ EVD	222 (67.5)	106 (59.2)	116 (77.3)	<**0.001**
Clinical outcome				
■ Duration of hospital stay (days), mean (±SD)	27.5 (11.6)	26.9 (12.2)	28.3 (10.8)	0.27
■ Follow-up (months), mean (±SD)	6.2 (2.9)	6.5 (2.3)	5.8 (3.4)	0.05
■ mRS at follow-up, median (IQR)	1 (0–4), *n* = 288	0 (0–3), *n* = 143	3 (0–5), *n* = 145	<**0.001**

ACA: anterior cerebral artery; BMI: body mass index; EVD: external ventricular drain; ICA: internal carotid artery; ICH: intracerebral hemorrhage; IQR: interquartile range; MCA: middle cerebral artery; mRS: modified Rankin Scale; SD: standard deviation.

Boldface type indicates statistically significant values.

### Time metrics and risk factors

**Figure 1.**
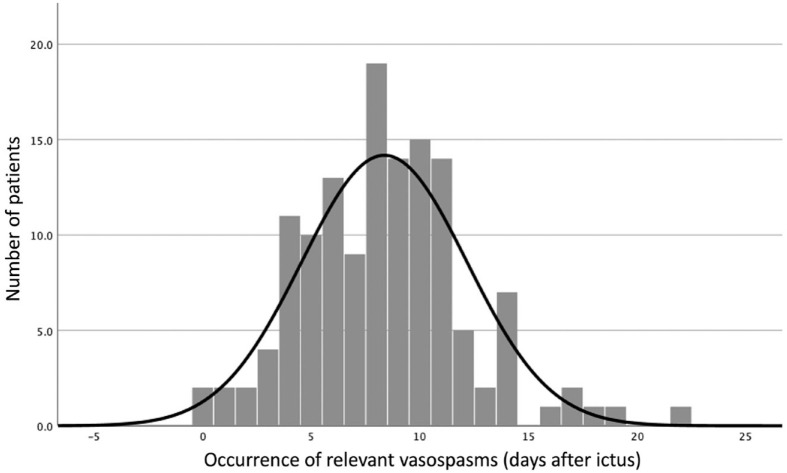
Distribution of onset of relevant vasospasms over time.

The median time until detection of relevant vasospasms was 8 days (IQR 6–10 days) (Figure 1). The median duration of relevant vasospasms was 2 days (IQR 1–5) and the median number of endovascular treatments per patient was two (IQR 1–3). The median time between the initial hemorrhage and last occurrence of relevant vasospasms was 11 days (IQR 8–14).

Further, we divided the patients into two groups: (1) patients with early onset of vasospasms and (2) patients with late onset of vasospasms. The threshold used to divide the two groups was the median onset time of 8 days. Early vasospasms were detected in 53 patients and late vasospasms in 82 patients, respectively. Patients who presented with new infarctions at discharge but without the detection of vasospasms were excluded from this analysis. Patients with early vasospasms were significantly younger (52.7 ± 11.2 years vs 58.7 ± 11.5 years, *p* = 0.003) and had more vasospasm-associated infarctions at discharge (58.8% vs 38.7%, *p* = 0.03). There was no significant difference in mRS at follow-up between the two groups. The demographic and clinical characteristics of patients with early- and late-onset relevant vasospasms are compared in Table 2.

**Table 2.** Demographic and clinical parameters of patients with relevant vasospasms.

	Early vasospasms *n* = 53	Late vasospasms *n* = 82	*p*-Value
Baseline characteristics			
■ Age (years), mean (±SD)	52.7 (11.2)	58.7 (11.5)	**0.003**
■ Female sex, n (%)	34 (64.2)	59 (72.0)	0.35
■ BMI (kg/m²), mean (±SD)	26.2 (4.3)	26.6 (5.2)	0.70
■ Diabetes, n (%)	1 (1.9)	1 (1.2)	1.00
■ Hypertension, n (%)	20 (37.7)	42 (51.2)	0.16
■ Smoker, n (%)	17 (32.1)	31 (37.8)	0.58
Aneurysm location, n (%)			
- *Anterior circulation*			
■ ICA	15 (28.3)	18 (22.0)	0.7
■ MCA	7 (13.2)	16 (19.5)	0.34
■ ACA	23 (43.4)	38 (46.3)	0.74
- Posterior circulation	12 (22.6)	14 (17.1)	0.42
Imaging details			
■ Modified Fisher grade, median (IQR)	4 (2–4)	4 (3–4)	0.62
■ ICH, n (%)	21 (39.6)	35 (42.7)	0.86
■ Vasospasm-associated infarction, n (%)	30 (58.8) (*n* = 51)	29 (38.7) (*n* = 75)	**0.03**
Clinical deficit			
■ Hunt and Hess grade, median (IQR)	3 (2–4)	3 (2–4)	0.78
Treatment details, n (%)			
■ Clip	12 (22.6)	26 (31.7)	0.25
■ Endovascular	41 (77.4)	57 (69.5)	0.32
■ EVD	40 (75.5)	62 (75.6)	1.00
Clinical outcome			
■ mRS at follow-up, median (IQR)	3 (0–5), *n* = 51	3 (0–4), *n* = 82	0.36

ACA: anterior cerebral artery; BMI: body mass index; EVD: external ventricular drain; ICA: internal carotid artery; ICH: intracerebral hemorrhage; IQR: interquartile range; MCA: middle cerebral artery; mRS: modified Rankin Scale; SD: standard deviation.

Boldface type indicates statistically significant values.

### Logistic regression analyses

After performing a multivariable logistic regression analysis for the occurrence of relevant vasospasms, younger age and additional ICH increased the odds for the occurrence of relevant vasospasms (OR 0.976, 95% CI [0.957, 0.995] and OR 1.78, 95% CI [1.022, 3.087]), while the other factors (sex, Hunt and Hess grade, modified Fisher grade, treatment modality, hypertension, smoker, diabetes mellitus, necessity of an EVD) showed no significant effect.

In a multivariable logistic regression analysis for early vasospasms, only age contributed significantly to predicting early vasospasms in the regression model (OR 0.949, 95% CI [0.914, 0.985]), while the other factors (sex, Hunt and Hess grade, modified Fisher grade, ICH at admission, hypertension, smoker, diabetes mellitus, necessity of an EVD) had no significant effect.

**Figure 2.**
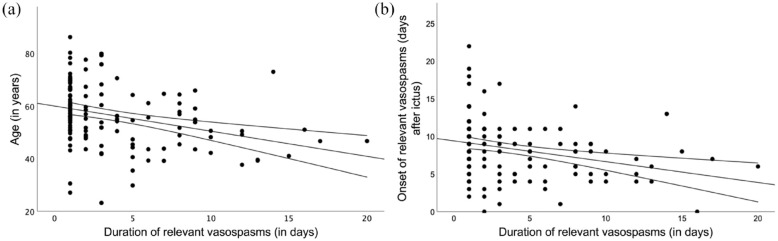
Dependence of the duration of relevant vasospasms on (a) patient age and (b) day of the occurrence of vasospasms.

As stated before, the median duration of relevant vasospasms under endovascular treatment was 2 days (IQR 1–5 days) ranging from 1 to 20 days. In 61 (45.2%) patients relevant vasospasms were detected at only one timepoint and did not recur after endovascular treatment. Multiple regression analysis was performed to predict the duration of relevant vasospasms from age, sex, ICH at admission, modified Fisher grade, Hunt and Hess grade, onset of relevant vasospasms, the necessity of an EVD, smoking, and hypertension. These variables statistically significantly predicted duration of relevant vasospasms, *F*(11, 121) = 3.101, *p* < 0.001, *R*^2^ = 0.22. However, only age (β = −0.11, *p* < 0.001) and onset of relevant vasospasms (β = −0.26, *p* = 0.005) added significantly to the prediction (Figure 2). For a detailed overview of each regression model see Supplemental Figure 2.

Fifteen patients presented vasospasm-related infarctions at discharge in CT or MRI. In these patients vasospasms were not detected applying our in-house diagnostic algorithm. On closer retrospective inspection of these cases, 11 patients showed neither clinical symptoms suggesting relevant vasospasms nor elevated TCD measures. Three patients did not have increased TCD measures but could not be clinically monitored due to intubation and sedation. Two of the 15 patients presented at least at one time point with a focal neurological deficit (both with one-sided upper limb weakness) but had no other suspect findings (neither elevated TCD nor CTA showing vessel narrowing) confirming relevant vasospasms.

## Discussion

In this retrospective observational study, we described the timely occurrence and duration of relevant vasospasms after aSAH and investigated risk factors associated with early or prolonged vasospasms. A total of 150 (45.6%) patients were diagnosed with relevant vasospasms according to our local diagnostic protocol or by new infarctions suspicious for vasospasms in images at discharge. Previous studies have reported an incidence of cerebral vasospasms between 9% and 93%.^1^ The broad range of reported incidences might be due to the non-uniform definition of cerebral vasospasms. Most studies used CTA, DSA or elevated flow profiles in TCD to confirm cerebral vasospasms. Reviewing the current literature, a total of 41 different definitions of cerebral vasospasms were depicted.^1^ We used our local diagnostic protocol to diagnose cerebral vasospasms, which also includes the presence of new neurological deficits or pathological brain perfusion in intubated and sedated patients, beside daily TCD controls and CTA. Further, cerebral vasospasms were confirmed in DSA. We, therefore, refer to relevant vasospasms in our study, which we believe tend to represent a higher risk for cerebral infarction.

While age, Hunt and Hess grade, modified Fisher grade, necessity of an EVD due to imminent hydrocephalus and presence of ICH at admission differed significantly between patients who developed relevant vasospasms and patients who did not, only younger age and presence of ICH were significant prognostic factors in the regression analysis for developing vasospasms. Wan et al. found similar results in their study of 5362 SAH patients, showing that concurrent ICH is a risk factor for cerebral infarction after aSAH. The results imply that additional ICH may be a stronger predictor of cerebral vasospasms than commonly used parameters such as hypertonus, aneurysm characteristics, and the modified Fisher grade.^11^ Likewise, Platz et al.^12^ showed that ICH presence is associated with a higher risk of DCI, highlighting its prognostic significance at admission alongside the conventional modified Fisher grade. However, patients with additional ICH on admission had a higher Hunt and Hess grade compared to patients without ICH (median and IQR of Hunt and Hess grade for patients with ICH: 3 (2–4); and patients without ICH: 1 (1–3)). Therefore, people with ICH were more difficult to evaluate clinically and received CTA and CTP more often, leading to a higher detection rate of vasospasms.

We also missed to detect relevant vasospasms in 15 patients, who presented new infarction at discharge which were not present in images after treatment. It is not rare for aSAH patients to develop infarctions without detectable vasospasms. It has been speculated that these infarctions occur due to vasospastic reaction of the most distal superficial and intraparenchymal vessels which are difficult to detect in TCD or CTA and might have been missed by our diagnostic algorithm.^13^ The early detection and prevention of these infarction will remain a future challenge.

Consistent with previous findings,^14^ the median time from hemorrhage to vasospasms in our study was 8 days, ranging from 0 to 22 days.

While younger age has been reported as a risk factor for cerebral vasospasms overall,^15^ our data suggest that it is also a risk factor for their early development. Even more interestingly, patients with early vasospasms had a higher percentage of vasospasm-associated infarctions at discharge. Schmidt et al.^16^ presented similar results, demonstrating that early-onset DCI before day 7 was associated with higher mortality due to more severe infarct load. Early-onset vasospasms potentially lead to prolonged ischemia, subsequently causing more detrimental effects.^17^ Hence, it seems reasonable to closely monitor for potential vasospasms in the first week after aSAH. This involves, for example, increasing the frequency of TCD measurements and clinical reassessment from once to twice daily. It can be discussed whether CTA or CTP is an appropriate diagnostic tool to differentiate cerebral vasospasms in younger patients.^18^ CTA and CTP only capture a snapshot in time and offer limited utility as a screening test due to their poor sensitivity.^19^ Furthermore, the radiation exposure in younger patients should be considered critically.^20^

Unlike prior findings, our results do not associate female sex with altered risk for vasospasms.^21,22^ This could be due to our definition of relevant vasospasms and our in-house diagnostic algorithm, which does not routinely use CTA, CTP or DSA to screen for vasospasms. For example, Lai et al.^22^ routinely performed CTA or DSA in all patients with aSAH within 14 days regardless of symptoms and found an overall higher number of patients with radiographic vasospasms.

The median duration of relevant vasospasms under endovascular therapy was two days. Younger age and early onset of vasospasms were associated with a longer duration of relevant vasospasms and a higher rate of endovascular treatments. This highlights the importance of closely monitoring young patients with early signs of relevant vasospasms. However, whether endovascular therapies, such as intra-arterial infusion of nimodipine or balloon angioplasty are sufficient in reducing vasospasms and improving outcomes after aSAH remains debated.^23,24^

Patients with relevant vasospasms or vasospasm-associated infarctions presented with worse functional outcome at follow-up. However, we observed no difference in functional outcomes between patients with early versus late vasospasms. Of note, it was not the intention of this study to investigate risk factors associated with poor functional outcome and the overall conclusion is limited due to the incomplete clinical data and the retrospective study design. Additionally, it is challenging to distinguish between prognostic factors and possible treatment effects of endovascular procedure as randomized trials focusing on the impact of such therapies are still missing.

### Limitations

The present study contains all limitations that come along with a retrospective study design. As mentioned before, there is no uniform definition of relevant cerebral vasospasms. Therefore, it is challenging to compare our results with similar studies who used different inclusion and exclusion criteria for diagnosing and treating vasospasms endovascularly. Also, our algorithm to detect cerebral vasospasms might differ from other institutions and focuses on neurological examinations and TCD to screen for vasospasms. We did not perform routinely CTA or CTP in conscious patients, which is still a point of critical debate. Our definition differs from the definition of DCI by Vergouwen et al.^10^ which sets a focus on clinical deterioration rather than radiographic vasospasms. We used the term “relevant vasospasms” as it is more commonly used in the radiological setting and relevant for treatment decisions, such as intra-arterial spasmolytic treatment or angioplasty. Finally, we had no uniform imaging modality at discharge and patients with detected relevant vasospasms were less likely to receive MRI. This could have led to an underestimation of infarctions in this group.

## Conclusion

Our findings indicate a compelling relationship between younger age and the incidence of relevant vasospasms following aSAH. Furthermore, our study highlights that this correlation extends to the occurrence of early and prolonged vasospasms. This underscores the importance of increasing neurological monitoring in younger patients with signs of relevant vasospasms. Further prospective studies are warranted to determine whether prolonged endovascular treatment has a negative impact on outcome.

## Supplemental Material

sj-pdf-1-eso-10.1177_23969873231209819 – Supplemental material for Early and recurrent cerebral vasospasms after aneurysmal subarachnoid hemorrhage: The impact of age Supplemental material, sj-pdf-1-eso-10.1177_23969873231209819 for Early and recurrent cerebral vasospasms after aneurysmal subarachnoid hemorrhage: The impact of age by Bogdana Tokareva, Lukas Meyer, Christian Heitkamp, Rabea Wentz, Tobias D Faizy, Hanno S Meyer, Maxim Bester, Jens Fiehler and Christian Thaler in European Stroke Journal
