# Supplementary material for: Early and recurrent cerebral vasospasms after aneurysmal subarachnoid hemorrhage: The impact of age
Source: Eur Stroke J. 2023 Nov 1;9(1):172–9. doi: 10.1177/23969873231209819 (PMC10916818; doi:10.1177/23969873231209819)
Supplement: sj-pdf-1-eso-10.1177_23969873231209819 – Supplemental material for Early and recurrent cerebral vasospasms after aneurysmal subarachnoid hemorrhage: The impact of age [file sj-pdf-1-eso-10.1177_23969873231209819.pdf]

Supplementary Material

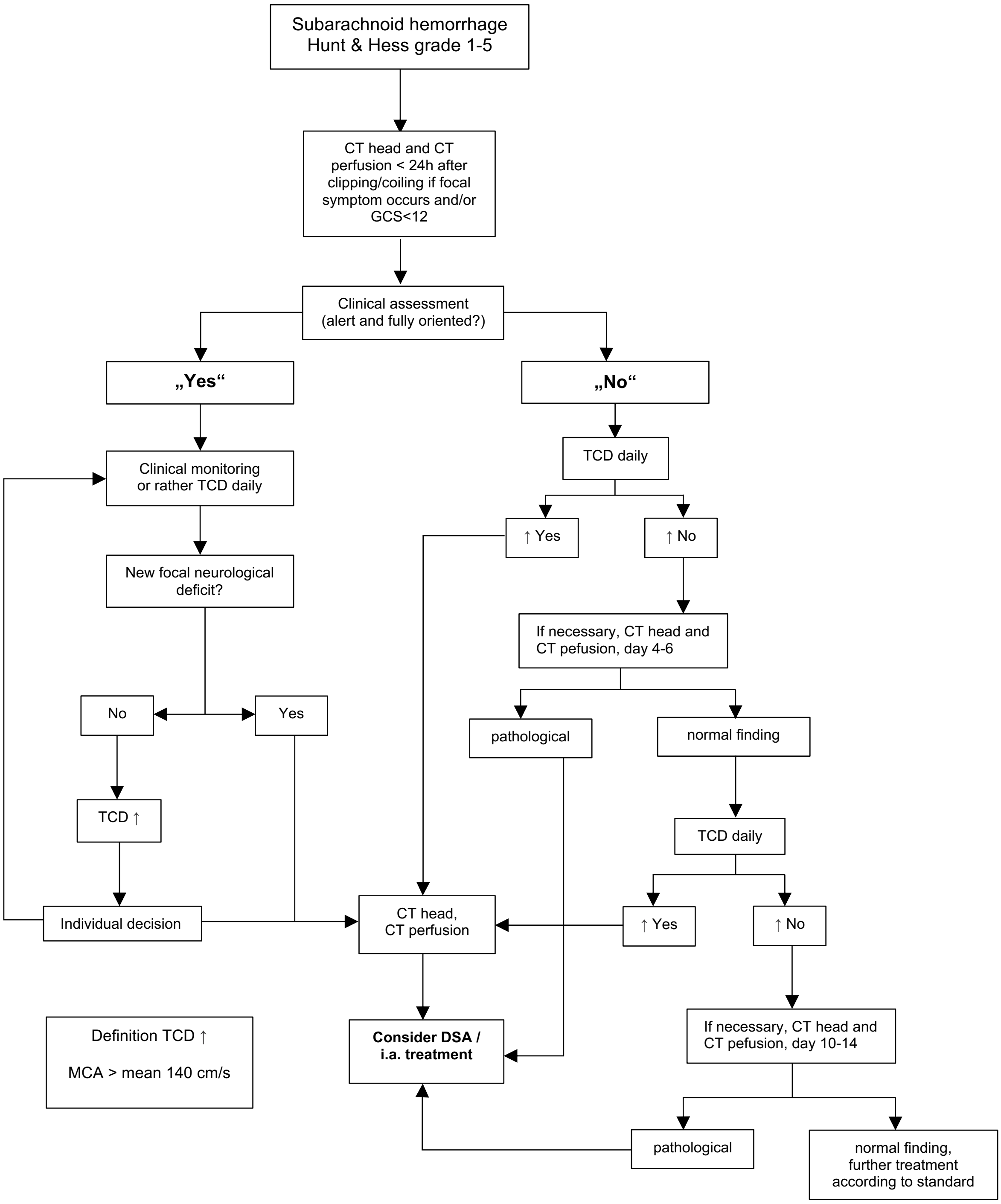

Suppl. Figure 1. Local protocol of diagnostic algorithm for detection of relevant vasospasms after subarachnoid hemorrhage.

CT computed tomography, *DSA* digital subtraction angiography, *GCS* Glasgow Coma Scale, *MCA* middle cerebral artery, *TCD* transcranial Doppler

	Model 1			Model 2			Model 3		
Variables	OR	95% CI	P Value	OR	95% CI	P Value	Coefficients	95% CI	P Value
Age (years)	**0.976**	**0.957 - 0.995**	**0.014**	**0.949**	**0.914 - 0.985**	**0.006**	**-0.109**	**-0.17 - 0.048**	**<0.001**
Sex (female)	1.446	0.872 - 2.398	0.15	0.563	0.246 - 1.290	0.174	-0.948	-2.407 - 0.51	0.20
Hypertension	0.69	0.42 - 1.133	0.14	1.095	0.489 - 2.449	0.826	0.565	-0.834 - 1.965	0.43
Diabetes m. (yes)	1.002	0.140 - 7.146	1.00	-	-	-	-2.577	-10.248 - 5.095	0.51
Smoking (yes)	0.755	0.459 - 1.244	0.27	1.705	0.734 - 3.958	0.215	-0.161	-1.623 - 1.301	0.83
ICH at admission (yes)	**1.78**	**1.022 - 3.087**	**0.04**	0.789	0.328 - 1.90	0.598	0.102	-1.431-1.634	0.90
EVD (yes)	0.555	0.291 - 1.057	0.07	1.070	0.367 - 3.119	0.901	0.662	-1.244 - 2.568	0.49
Hunt & Hess grade (increasing)	1.088	0883 - 1.340	0.43	1.129	0.808 - 1.577	0.477	-0.114	-0.704 - 0.476	0.70
modified Fisher grade (increasing)	1.170	0.904 - 1.513	0.23	0.847	0.554 - 1.297	0.446	0.367	-0.371 - 1.105	0.33
Treatment modality (Clip)	0.755	0.47 - 1.416	0.47	1.648	0.694 - 3.917	0.258	-1.038	-2.506 - 0.43	0.16
Onset of relevant vasospasms	na			na			**-0.255**	**-0.432 - 0.077**	**0.005**
Constant	-	-	-	-	-	-	12.100	2.126	<0.001

Suppl. Figure 2. Binomial and multivariate regression models.

Boldface type indicates statistically significant values.
EVD external ventricular drain, *ICH* intracerebral hemorrhage

Model 1: Binomial logistic regression, dependent variable “occurrence of relevant vasospasms”
Model 2: Binomial logistic regression, dependent variable “occurrence of early relevant vasospasms”
Model 3: Linear regression, dependent variable “duration of relevant vasospasms”
NA: Not selected in the model
